# How does sanitation influence people's quality of life? Qualitative research in low-income areas of Maputo, Mozambique

**DOI:** 10.1016/j.socscimed.2021.113709

**Published:** 2021-03

**Authors:** Ian Ross, Oliver Cumming, Robert Dreibelbis, Zaida Adriano, Rassul Nala, Giulia Greco

**Affiliations:** a Department of Global Health and Development, London School of Hygiene and Tropical Medicine, 15-17 Tavistock Place, London, WC1H 9SH, United Kingdom; b Department of Disease Control, London School of Hygiene and Tropical Medicine, Keppel Street, London, WC1E 7HT, United Kingdom; c WE Consult, Tomás Ribeiro street 177, Maputo, Mozambique; d Instituto Nacional de Saúde, Distrito de Marracuene, Estrada Nacional 1, Maputo, Mozambique

**Keywords:** Health economics, Sanitation, Quality of life, Qualitative research, Toilets, Capability approach

## Abstract

Preventing infectious disease has often been the primary rationale for public investment in sanitation. However, broader aspects of sanitation such as privacy and safety are important to users across settings, and have been linked to mental wellbeing. The aim of this study is to investigate what people most value about sanitation in low-income areas of Maputo, Mozambique, to inform a definition and conceptual model of sanitation-related quality of life. Our approach to qualitative research was rooted in economics and applied the capability approach, bringing a focus on what people had reason to value. We undertook 19 in-depth interviews and 8 focus group discussions. After eliciting attributes of “a good life” in general, we used them to structure discussion of what was valuable about sanitation. We applied framework analysis to identify core attributes of sanitation-related quality of life, and used pile-sorting and triad exercises to triangulate findings on attributes’ relative importance. The five core attributes identified were health, disgust, shame, safety, and privacy. We present a conceptual model illustrating how sanitation interventions might improve quality of life via changes in these attributes, and how changes are likely to be moderated by conversion factors (e.g. individual and environmental characteristics). The five capability-based attributes are consistent with those identified in studies of sanitation-related insecurity, stress and motives in both rural and urban areas, which is supportive of theoretical generalisability. Since two people might experience the same toilet or level of sanitation service differently, quality of life effects of interventions may be heterogeneous. Future evaluations of sanitation interventions should consider how changes in quality of life might be captured.

## Highlights

• Different sanitation interventions might improve quality of life to varying degrees.
• We explored attributes of sanitation-related quality of life as capabilities.
• We identified five core attributes: health, disgust, shame, safety, and privacy.
• Results aligned with studies framed in terms of insecurity, stress and motives.
• Conversion factors moderate how sanitation (e.g. a toilet) supports capabilities.

## 1 Introduction

Preventing infectious disease has often been the primary rationale for public investment in sanitation, defined as the separation of human excreta from human contact (WHO, 2018). Approximately two billion people globally lack access to “basic” sanitation services, defined as an improved type of facility which is not shared with other households (UNICEF/WHO, 2019). An estimated 432,000 annual deaths from diarrhoeal disease are attributable to inadequate sanitation (Prüss-Ustün et al., 2019).

However, health is more than the absence of disease. It is “a state of complete physical, mental, and social well-being” (WHO, 1948). Sanitation also affects these broader aspects of health. A systematic review of the relationship between sanitation and mental well-being identified privacy and safety as root dimensions, predominantly based on qualitative studies (Sclar et al., 2018). Aspects underlying these dimensions were identified as shame, anxiety, fear, assault, dignity and embarrassment. Beyond mental well-being, cleanliness and convenience are also commonly reported as important by users (Novotný et al., 2018). Collectively, we denote these aspects emphasised by users as “quality of life attributes”. They are rarely measured in impact evaluations of sanitation programmes, which predominantly focus on disease (Wolf et al., 2018) and toilet use (Garn et al., 2017).

Sanitation cost-benefit studies have noted that improvement in quality of life (QoL) attributes would comprise an economic benefit with a monetary value, but that methods for incorporating them are lacking (Hutton et al., 2014, 2020; Whittington et al., 2020). Economists, then, see privacy, safety or dignity as *outcomes* with an economic value to individuals, but have not attempted to measure them. Sanitation-focused research from other disciplinary perspectives has approached these issues in other ways. The most widely-cited work on QoL attributes has studied them as motives, for example by aiming to identify the behavioural drivers of open defecation (Jenkins and Curtis, 2005; Jenkins and Scott, 2007; Mukherjee, 2001). With disciplinary roots in psychology, these studies see safety for example as a driver of a decision, rather than as an outcome which different interventions might improve to different degrees (Aunger and Curtis, 2013).

Another group of studies assesses QoL attributes as sources of stress and insecurity, often with an epidemiological orientation focused on mental wellbeing outcomes (Caruso et al., 2017b; Kwiringira et al., 2014; Sahoo et al., 2015; Shiras et al., 2018). For example, a measure of women's sanitation insecurity includes aspects of privacy, safety, and so on (Caruso et al., 2017a). It has been used in evaluative studies as a risk factor (Caruso et al., 2018) or effect moderator (Delea et al., 2019) on the causal pathway to mental well-being. In other words, women's sanitation insecurity is applied as an explanatory variable, rather than an outcome in itself.

In the sanitation sector, then, QoL attributes have not been seen as outcomes to be measured and valued, but this is not the case in other sectors. Health-related QoL, for example, is routinely measured in health impact evaluations and applied in cost-effectiveness studies (Karimi and Brazier, 2016). Economic approaches to conceptualising QoL often have the ultimate aim of informing the allocation of public funds, typically leading them to be broadly framed so as to apply to the general population (Fayers and Machin, 2015). Applying an economic perspective to the impact of sanitation on QoL requires a focus on value, or what is important to people. There are divergent traditions within economics on the conceptualisation of value. In utilitarian welfare economics, value is defined by an individual's subjective utility, or the satisfaction they derive from goods or activities (Stiglitz et al., 2009). The capability approach to welfare economics, meanwhile, considers utility problematic due to its focus on individuals' psychological states which can adapt to experience and expectations (Sen, 1980, 1993). Under the capability approach, a good life comprises what people are *able* to do and to be, with QoL attributes identified by discussion of what people “have reason to value” (Sen, 1999). “Conversion factors” are the degree to which an individual can convert “commodities” into capabilities (Robeyns, 2005). An individual has the choice of whether to actually act on those capabilities as “functionings”, making the evaluative space an individual's capability to function.

Therefore, while a utilitarian approach to sanitation-related QoL might focus on satisfaction, a capability approach would focus on what people are able to be and do with respect to their sanitation practices. This frames sanitation facilities and services as commodities in support of sanitation-related capabilities. We know of only one peer-reviewed paper applying capabilities to sanitation, a review rather than an empirical study (Barrington et al., 2017). Health economic studies are increasingly using the capability approach to inform the development of outcome measures, based on qualitative research (Al-Janabi et al., 2012; Canaway et al., 2017; Greco et al., 2015; Grewal et al., 2006; Kinghorn et al., 2014).

In assessing the impact of sanitation on QoL, all sources of value can be considered, not only avoiding negative outcomes such as stress or insecurity. Furthermore, in economic applications the *relative* value of different attributes is important. We know of eight studies which provided or enabled a user-reported ranking of motives, stressors or benefits related to sanitation (Gross and Günther, 2014; Hulland et al., 2015; Jenkins, 1999; Jenkins and Curtis, 2005; Jenkins and Scott, 2007; Kiyu and Hardin, 1993; Lagerkvist et al., 2014; Mukherjee, 2001). Only one was in a predominantly urban setting, which was quantitative and focused on motives for use of a “peepoo” bag, not improved sanitation (Lagerkvist et al., 2014).

The aim of this study is to investigate what people most value about sanitation in a low-income urban setting, to inform a definition and conceptual model of sanitation-related quality of life. We use qualitative methods to examine how sanitation contributes to “a good life”, by analysing the accounts of users of different types of shared toilet facilities in informal settlements in Maputo, Mozambique. Our underlying objective is to inform the development of a quantitative psychometric measure of sanitation-related QoL.

## 2 Methods

We applied a variety of methods to identify and explore attributes of sanitation-related quality of life in the broad evaluative space of capabilities. We used interviews to obtain in-depth accounts, and focus groups to ensure a broader range of views were considered, as well as to engender the deliberation encouraged by the capability approach. We also used pile-sorting and triads, which are structured data collection approaches from cognitive anthropology, to triangulate findings on attributes’ relative importance (Weller and Romney, 1988). We followed the consolidated criteria for reporting qualitative research (COREQ) guidance (Tong et al., 2007), with reporting summarised in Supplementary Material A.

### 2.1 Study setting and intervention

This study was linked to the Maputo Sanitation trial (MapSan), which evaluated an urban shared sanitation intervention (clinicaltrials.gov registration: NCT02362932) (Knee et al., 2020). We used the trial as a vehicle, but our study is not an evaluation of the intervention. In Mozambique, 48% of urban residents lack access to basic sanitation (UNICEF/WHO, 2019). Maputo City, Mozambique's capital, has a population of 1.1 million people (INE, 2019) of which 70% live in informal settlements (UN-HABITAT, 2010). Non-sewered sanitation facilities are used by 89% of Maputo residents, and only 26% of fecal waste in the city is safely managed (Hawkins and Muximpua, 2015). The MapSan trial took place in the low-income *bairros* (neighbourhoods) of the Nhlamankulu district, where multi-household “compounds” with a single entrance to a shared courtyard are common.

People carry out their sanitation practices in a part of the compound called the *casa de banho.* We translate this as “toilet”, in the international english sense of meaning any sanitation facility. When referring to specific technologies, we denote those *with* a water-seal (“U-bend”) as pour-flush toilets and those *without* as pit latrines (Cairncross and Feachem, 1983). We considered the scope of sanitation practices as perceived by participants, noting that the *casa de banho* (toilet) space is used for bathing and menstrual hygiene management in addition to defecation and urination (Shiras et al., 2018). Before the intervention evaluated by MapSan, toilets mostly comprised an informally-fenced space containing a pit latrine, shared with other households on the compound. Most pit latrines were “traditional”, with a soil floor (photos in Supplementary Material B). The intervention was delivered during 2015-16 by Water and Sanitation for the Urban Poor (WSUP), an international non-governmental organisation (NGO), with users making a 10–15% financial contribution. MapSan intervention compounds were provided with a flush or pour-flush toilet discharging to a septic tank, shared with other households on the compound as before. There were two superstructure designs depending on the number of users, all stand-alone buildings not connected to any house. Control compounds continued to use shared pit latrines. We provide more information in about the intervention in Supplementary Material B in the TIDieR checklist format (Hoffmann et al., 2014).

### 2.2 Field team and sampling strategy

The field team comprised four interviewers aged 25–40, two male and two female, led by ZA. All interviewers spoke fluent Portuguese and Changana, the first and second most commonly spoken languages in Maputo. Interviewers underwent a week of training and piloting with IR and ZA which covered informed consent, interview techniques, and transcription skills. Each interviewer undertook interviews and focus group discussions in the setting, observed by IR and ZA, followed by daily team debriefings. Interviewers were from various parts of Maputo and none were known to participants.

During November–December 2018 we conducted 19 interviews and eight single-sex focus groups of 4–8 participants. The same sampling strategy was followed for interviews and focus groups. To limit respondent fatigue amongst the MapSan study population who had already participated in other trial-related research, we recruited from its broader target population. Specifically, this was multi-household compounds in the same bairros, some of whom had received the same NGO intervention. This meant our study population used the same toilet types as the MapSan intervention and control groups. Our sampling strategy was stratified by three characteristics. We aimed for approximately equal numbers of female and male participants, as well as a mix of respondents by age (18–24, 25–59 and 60+) and toilet type used. We recruited participants by going door-to-door, based on the NGO's records of multi-household compounds, and sampled purposively until a mix of people across strata was achieved. Due to the relatively small sample size, this strategy did not aim to enable exploration of differences between sub-groups, but rather to ensure findings were influenced by a breadth of experiences. Focus groups were convened by the gender and age strata – one was exclusively women aged 18–24, and so on. The majority of interviews and focus groups took place in Portuguese. Changana was used, mostly in short sections, in 16% of transcripts. Interviews and focus groups were audio-recorded, transcribed in Portuguese and translated into English. Interviewers emphasised to participants that they were independent researchers and not working for the implementing NGO or local government.

### 2.3 Data generation and topic guides

**Fig. 1:**
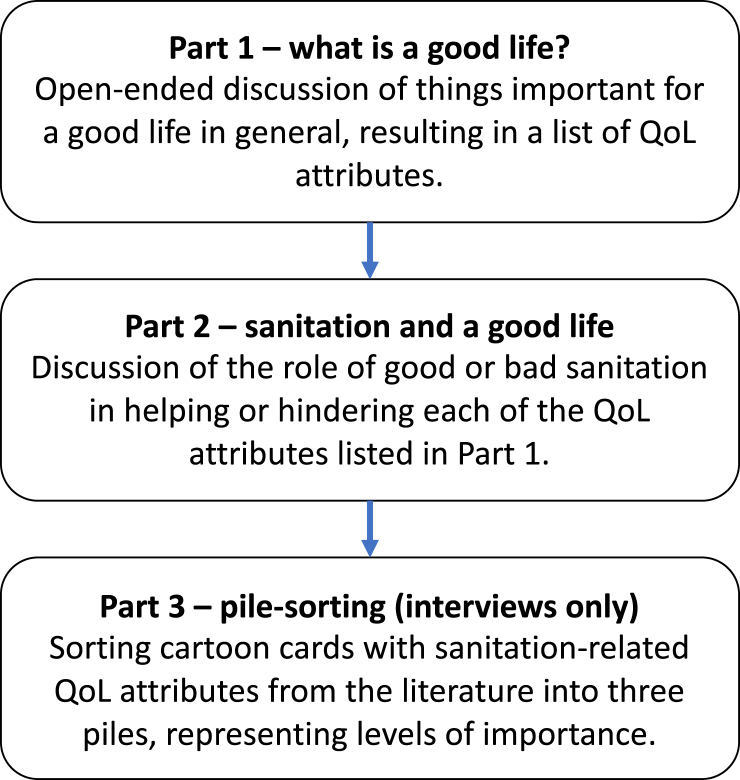
Structure of interview and focus group topic guides.

One interviewer of the same gender as the participant carried out each interview, which lasted on average 40 min. The interview topic guide, adapted iteratively during training and piloting, is included in Supplementary Material C and summarised in Fig. 1. Part one aimed to identify valued attributes of a good and bad life in general. Part two discussed the role of sanitation in affecting those valued attributes (Fig. 1) considering all sanitation practices important to participants. Part three used pile-sorting (Weller and Romney, 1988), which generated structured data rather than a verbal account. Participants were presented with 15 cards, each with an attribute of good sanitation identified in previous reviews (Jenkins and Sugden, 2006; Novotný et al., 2018). Cards comprise the bar labels in Fig. 4 and included cartoon depictions (Supplementary Material C). Participants were asked to choose the five that they thought were most important for a good life, then a second set of five for the next level of importance. The remaining five cards comprised the third set.

Focus groups were managed by one moderator and one notetaker, of the same gender as participants. Each lasted on average 60 min. The first six focus groups followed the same guide as interviews, but omitting the pile-sorting exercise in the interests of time and practicality. For the final two focus groups (one male, one female), we reconvened 6–8 participants from previous focus groups and interviews. First, emerging findings were presented and reflections sought in a “participant checking” discussion (Green and Thorogood, 2009), then we proceeded with a triad exercise (Weller and Romney, 1988). Participants were presented with three attributes and asked to choose the most important of them, and the process then repeated with different combinations. Attributes on triad cards were based on emerging analysis, and fewer in number than pile-sorting cards to reduce respondent fatigue (Supplementary Material D).

### 2.4 Data analysis

Value was the “economic lens” (Coast and Jackson, 2017) in design of data generation activities and subsequent analysis, specifically the capability approach and its focus on “reason to value” (Sen, 1999). The primary output of our analysis is a conceptual model comprising theory about what people value about sanitation, alongside contextual qualitative description. The focus on value and capabilities influenced our design and analysis in several ways. First, it meant we prioritised the exploration of the relative importance of attributes through multiple methods. Second, the topic guide was organised such that the evaluative space was broad, focused on “a good life”. Third, core attributes in the eventual conceptual model were framed as capabilities. Fourth, we coded passages which related to conversion factors, i.e. the characteristics of individuals and their physical and social environment.

We used ‘framework’ analysis to interpret transcripts (Ritchie and Spencer, 1994). Framework is not associated with a specific epistemological position, and allows for both inductive and deductive coding (Green and Thorogood, 2009; Ritchie and Lewis, 2003). We followed an interpretive approach, acknowledging that data based on interaction in interviews and focus groups only describes one version of reality (Coast et al., 2017). We followed the five steps of framework analysis (Ritchie et al., 2003) to understand the data: (i) familiarisation; (ii) identifying a thematic framework; (iii) indexing; (iv) charting; and (v) mapping and interpretation. As part of step 1, after ZA had verified transcription and translation, IR uploaded them into nVivo 12 (QSR International, 2018) and wrote a summary memo of each. Transcripts were presented in English and Portuguese paragraph by paragraph, enabling coding of the English version with easy reference to the original Portuguese where necessary. For step 2, coding took an inductive approach, without applying any *a priori* codes. IR, who understands Portuguese, open-coded four interview transcripts as an initial batch and discussed the resulting codes with GG. Closely-related codes were combined and redundant codes deleted. The codebook was applied to the next batch of four, the process repeated, and a framework gradually emerged. The codebook was rarely updated after the third iteration, suggestive of theoretical saturation. The final codebook (“index”) was then applied to remaining interviews and all focus groups (step 3). Based on charting (step 4), IR wrote analytical memos for the most salient concepts, in support of interpretation (step 5).

Through this process, we arrived at a set of “core attributes” which are sanitation-related capabilities, and several “underlying concepts” for each. We would highlight three aspects of the process. First, core attributes incorporated both positive and negative aspects of the same concept (e.g. pride and shame) and linked concepts (e.g. smelling faeces and seeing maggots). Second, in refining codes, we built on Al-Janabi's (2012, p. 169) approach, to reflect “less the specific influences on well-being (e.g. work) and more the concepts that could be influenced by multiple factors (e.g. stress). … that represented what was ultimately important in individuals' lives”. Third, we aimed to be reflexive in our analytical processes of identifying and applying a framework, considering alternative ways in which concepts could be labelled and codes aggregated. To the extent possible, codes reflected the *in vivo* phrases used by respondents in Portuguese. However, it is impossible to exclude the role of the researcher in shaping analysis.

Core attributes were identified only through the above analysis of transcripts, with other data analysis used only for triangulation. After core attributes were identified, we added intensity codes to interview excerpts coded with those attributes (Saldaña, 2015). We did not do this for focus groups, since the relative tone attached to different passages is more uncertain when many people are contributing and interacting. Passages were coded as “low intensity” when participants mentioned the topic in passing or in a short impassive phrase, “medium” when discussed in more detail or with mild emotion, or “high” when a lengthy example or emotive language was used. We used nVivo coding queries to map intersections of intensity codes with content codes, and tabulated frequencies. In analysis of pile-sorting data, concepts in the top pile were given three points (two in sensitivity analysis), the second pile one point, and the remaining pile zero points.

### 2.5 Ethical considerations

All participants provided written informed consent to participate. We informed participants of their right to end discussions at any time, and all audio recordings were permanently deleted after verification of transcripts. The study received approval from the Research Ethics Committee of the London School of Hygiene and Tropical Medicine (Ref: 14609) and from the *Comité Nacional de Bioética para a Saúde* (IRB00002657) at the Ministry of Health in Mozambique.

## 3 Findings

There were 73 participants in the study overall, of which 41 were women and 32 men (Table 1). Most participants (75%) used a toilet shared by 2–5 households. There were more users of flush toilets than pit latrines, but the majority of these had received the NGO intervention in the past 1–2 years so could reflect on pre-intervention experience with pit latrines. Two female interview participants were pregnant, and two male interview participants had a physical disability affecting their mobility.

**Table 1:** Characteristics of participants.

	Interviews % and (n)	Focus groups % and (n)	Overall % and (n)
**Gender**			
Female	53% (10)	57% (31)	56% (41)
Male	47% (9)	43% (23)	44% (32)
**Age**			
18-24	37% (7)	37% (20)	37% (27)
25-59	42% (8)	50% (27)	48% (35)
60+	21% (4)	13% (7)	15% (11)
**Education**			
Did not complete primary	26% (5)	41% (22)	37% (27)
Primary or incomplete secondary	42% (8)	48% (26)	47% (34)
Completed secondary or above	32% (6)	11% (6)	16% (12)
**Tenancy**			
Owners	79% (15)	76% (41)	77% (56)
Renters	21% (4)	24% (13)	23% (17)
**Toilet type**^a^			
NGO-supported flush/pour-flush	47% (9)	17% (9)	25% (18)
Other flush/pour-flush	16% (3)	46% (25)	38% (28)
Pit latrine	37% (7)	37% (20)	37% (27)
**Households using toilet**			
1 (private)	0% (0)	13% (7)	10% (7)
2-5	79% (15)	74% (40)	75% (55)
6+	21% (4)	13% (7)	15% (11)

a As set out in the methods section, we recruited from the target population of the MapSan trial, not its study population. Users of “NGO-supported flush/pour-flush” are analogous to the MapSan intervention group, and users of “pit latrine” are analogous to the control group.

Below we present findings for each of five capabilities which are core attributes of sanitation-related QoL, representing what participants most valued about sanitation in this setting: health, avoiding disgust, avoiding shame, safety, and privacy. We provide illustrative quotations for each (Portuguese in Supplementary Material E). Afterwards, we present a conceptual model illustrating how the attributes fit together, and findings from the triangulation analyses. In participant checking undertaken in the final two focus groups, no concerns or proposals were raised about findings emerging at that time.

### 3.1 Health

The two main concepts underlying the health (*saúde*) attribute were disease (*doença*) and peace of mind (*paz de espírito*), Almost all interview participants (18/19) mentioned one or both, with roughly twice as many mentioning disease. Unspecified disease was most commonly cited, but specific symptoms (diarrhoea, vomiting) or pathogens (cholera) were too. There was general understanding that children touching faeces would spread disease, also emphasising concern for others as a relational aspect of QoL:

“It is difficult for us to control what children do. An adult knows they shouldn't touch something, or they'll catch germs, but a child doesn't know.” Male interview, 36 (EAGJ04)

Healthcare expenses as a result of disease were cited as a problem deriving from poor sanitation, and flooding was cited as a risk factor for sanitation-related diseases:

“When it rains the faeces in the pit rise up, then we get diseases because cholera comes from there” Female interview, 71 (EANC04)

The mental wellbeing aspect of health was most commonly framed as “peace of mind”, but also as feeling “at ease” (*a vontade*) and “relaxed” (*tranquilo*). It was far more often cited in relation to other attributes (disgust, shame, and privacy in that order) than on its own:

“You feel under pressure when the bathroom is dirty, and you don't feel at ease.” Female focus group, 18–24 (FGF01)

“Your neighbours will know the origin of the smell and will start to talk about it, and you can't feel relaxed.” Female interview, 76 (EAET04)

Peace of mind was also referred to positively, for example people with better-quality toilets reporting feeling relaxed while using it. It was sometimes mentioned without being linked to another specific concept:

“Having a good toilet contributes positively to all these aspects, mental health, wellbeing for the soul, and general health as a whole.” Male interview, 64 (EAGJ03)

### 3.2 Avoiding disgust

Two concepts underlying the disgust (*nojo*) attribute were sights/smells, and feeling clean/touch. Almost all interview participants (17/19) mentioned one or both, with sights/smells cited approximately twice as often. Seeing faeces was a common trigger of disgust for users of pit latrines, as they can often be seen through the drop-hole, but also for users of flush toilets which were not clean. Maggots, cockroaches and flies were also visual triggers of disgust for pit latrine users.

"It is something so horrible to see other people's faeces" Male interview, 19 (EAGJ02)

The toilet's smell was important both at the time of using it and at other times:

“You cannot eat because you lose your appetite … due to the smell. You don't even feel free to come out of your house because it smells bad out there.” Female focus group, 25–59 (FGF02)

When considering good toilets, people talked about positive consequences of a lack of disgust, for example being able to do more things in there rather than wanting to rush out immediately:

“[In this toilet] I feel like I am in the kitchen. With no bad smell, it seems like you could even drink tea in there, without realising you are in a toilet.” Female interview, 71 (EANC04)

Users of better-quality toilets reported appreciating their cleanliness, illustrating the positive side of this attribute. People would prefer to use a toilet that was clean, but also to *feel* clean both while there and after leaving (for example, after bathing).

“When the house is clean but the toilet is not, this is undignified.” Female focus group, 60+ (FGF03)

### 3.3 Avoiding shame

The two concepts underlying the shame (*vergonha*) attribute were “what others think or say” (pride/status), and dignity. Three quarters of interview participants (15/19) mentioned one, or more, and were roughly twice as likely to mention “what others think or say” as the other.

“A person's toilet becomes the mirror of that person.” Male interview, 64 (EAGJ03)

The most commonly reported trigger for shame was the disdain of others on account of the smell or appearance of the toilet. Shame could also be caused just by knowing that other people could smell the toilet, without even interacting with them:

“Everyone will refer to you according to the state of your toilet, saying ‘it's there at her house that the toilet smells’ … nobody respects you.” Female interview, 27 (EAET05)

“People who go down my road smell the stench from my toilet. Then when they later pass me on the street they will look at me in a different way.” Male interview, 19 (EAGJ02)

Several respondents with ‘good’ toilets reported feeling proud or feeling more respected by visitors, reflecting the positive side of this attribute:

“When I get visitors, I can let the person use the toilet without fear. I think this makes people look at me differently, with respect.” Male interview, 28 (EAJP05)

“If a visitor asks to go to the toilet and sees it in good condition, they'll say ‘wow, that lady's house is hygienic’” Female focus group, 60+ (FGF03)

### 3.4 Safety

The two concepts underlying the safety (*segurança*) attribute were accidents/falls, and violence (physical or sexual) which included “peeping”. Two thirds of interview participants (13/19) mentioned one or both, with accidents/falls reported roughly twice as frequently. For safety in general, prospective concern about things which *might* happen appeared much more prevalent than actual experience.

Respondents identified these risks with respect to themselves but also family members, again emphasising the relational aspect of QoL. For example, risk of injury was reported in relation to a child falling into the pit, or a poorly-constructed toilet collapsing:

“That toilet built from car tyres is a hazard – when it rains it could come crashing down at any moment.” Male focus group, 25–59 (FGM02)

Respondents with reduced mobility (e.g. pregnant women, older people, disabled people) were more likely to report fearing falling into the pit or falling over while squatting. With low-quality toilets, a risk was not being able to see properly at night:

“I'm afraid to use it at night because I wouldn't know which way to enter, where to tread inside, and I would be afraid of falling into the hole.” Female interview, 71 (EANC04)

Regarding violence, participants more often described the generalised risk of bandits and thieves. Sexual assault was seen as a risk for both men and women. People with high-quality toilets did not necessarily feel safer at night, because everyone needed to leave their house into an insecure compound to access the toilet building:

“There are people who are raped while they use these toilets. … there are times we even have to defecate in a bucket because we fear bandits.” Female interview, 27 (EAET05)

### 3.5 Privacy

The main concepts underlying the privacy (*privacidade*) attribute were being seen and being disturbed. Two thirds of interview participants (12/19) mentioned one or both, with “being seen” roughly four times as likely as being disturbed. Sometimes respondents knew people could see them over or through the walls or door, as they themselves could see passers-by or hear children laughing. However, there was also the fear that an unseen person might be “peeping” (see “safety” above). Privacy was important for all types of sanitation practices, including bathing and menstrual hygiene:

“You cannot imagine the gymnastics I do when I have my period. I do not feel relaxed because I do not know if I'm being watched.” Female interview, 27 (EAET05)

From the viewer's perspective, privacy could also be infringed unintentionally:

“While you walk to work, …you might see a woman with just a bit of capulana [fabric], when she is naked taking a bath.” Male interview, 28 (EAJP05)

The concept of being disturbed concerns someone else entering an unlocked or door-less toilet, without knowing you were inside.

“When a bathroom is not secure you do not feel free to use it because at any moment an individual can enter.” Female interview, 76 (EAET04)

Some respondents also mentioned a good-quality private toilet providing a place to do make-up, trim body hair, or be intimate with one's partner through showering together or sex.

### 3.6 Conceptual model

**Fig. 2:**
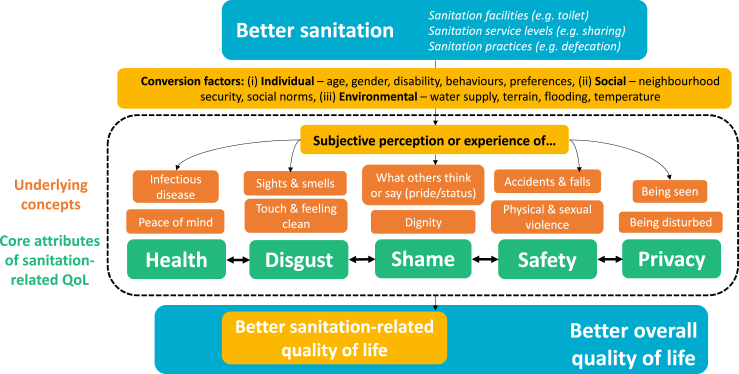
Conceptual model for sanitation-related QoL.

Fig. 2 presents a conceptual model for sanitation-related QoL, which visualises five findings. First, we define sanitation-related QoL as “the subset of overall QoL which is directly affected by sanitation practices or services”. This definition draws on analogous definitions of health-related QoL (Karimi and Brazier, 2016; Peasgood et al., 2014). The scope of sanitation practices is as perceived by users, but is assumed to include defecation, urination, menstrual hygiene, and any related practices users consider important. Second, five capabilities were identified as core attributes of sanitation-related quality of life (green in Fig. 2). Underlying each is a number of concepts (in orange). Third, an improvement in sanitation facilities, services or practices might cause an improvement in overall QoL, demonstrating the instrumental value of sanitation. That improvement might act via changed experience or perception around one or more of the capability-based attributes. Fourth, any effect of improving sanitation on QoL may be moderated by conversion factors. Examples include respondents with reduced mobility being more likely to fear falling into the pit, or people with good-quality toilets fearing using them at night. Based on our findings, we hypothesise that conversion factors include: (i) individual conversion factors, such as gender, age and disability, (ii) social conversion factors, such as neighbourhood security and social norms, (iii) environmental conversion factors, such as flooding and the level of the water table. Fifth, while these five attributes are distinct sources of value, they are also inter-related. Sometimes respondents’ safety concerns arose from privacy deficits, but many also related to the journey to the toilet through an insecure compound. A toilet being dirty or disgusting was perceived as bad regardless of the possible health consequences, which were mostly not mentioned as part of the same point. Shame was related to disgust and privacy, e.g. being embarrassed at using a smelly toilet or being seen.

### 3.7 Relative importance of attributes

**Fig. 3:**
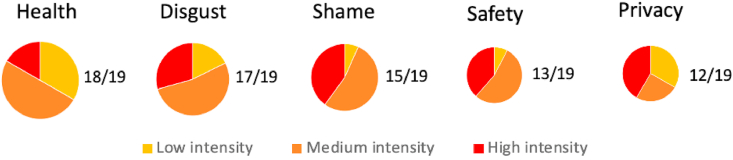
Frequency and intensity with which attributes were mentioned in interviews.

Participants discussed attributes with different frequency. In Fig. 3, the relative size of pies represents the proportion of interview participants mentioning each attribute at least once. Participants’ intensity of speech also varied – pie charts show the highest level of intensity used by each participant mentioning that attribute. This analysis shows that while health and disgust were mentioned most often, a lower proportion of participants mentioned them using highly intense phrases. This contrasts with shame and safety in particular, which were more likely to be mentioned in medium or high intensity language, showing the limitations of analysing frequency alone.

**Fig. 4:**
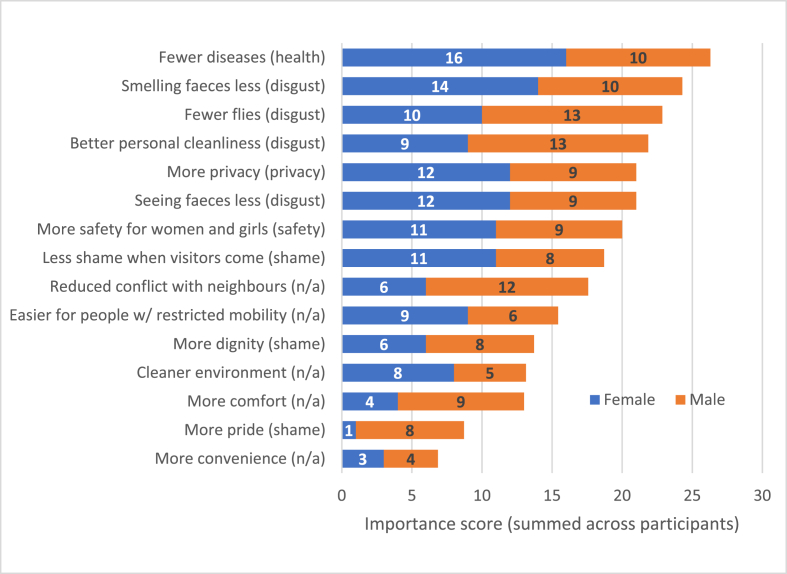
Importance scores from pile-sorting undertaken during interviews. *nb. labels (without bracketed part) were written on cards in Portuguese and read out by interviewers. n/a = does not map onto any single attribute.*

In the pile-sorting findings (Fig. 4), each bar shows the total of importance scores for 15 concepts, normalised to account for gender imbalance. Bar labels note the most closely-related core attribute in parentheses. Though the primacy of health and disgust is in line with frequency data presented above, pile-sorting data imply a slightly different ranking for the other attributes: (i) health, (ii) disgust, (iii) privacy, (iv) safety, (v) shame. Sensitivity analysis (two points instead of three for the first pile) did not change these findings. Health and disgust are again first and second in the triad data (Supplementary Material D), but the other attributes are again ordered slightly differently. This indicates uncertainty around the relative value of those three attributes for participants, given the small sample sizes involved. In ascertaining relative value, we would place more weight on the pile-sorting and triad data, where respondents directly traded off attributes. The frequency and intensity data (Fig. 3), by contrast, rely on our interpretation. Concepts which do not map onto any single attribute in our model are denoted “n/a” in bar label parentheses (discussed in Supplementary Material D). These concepts scored lower than the highest-ranked concepts linked to the five core attributes, and were in the bottom 40% of cards overall. There were some differences in scoring between sexes. The biggest absolute differences in scores were for pride, reduced conflict with neighbours (both of which men valued more) and fewer diseases (which women valued more). Women were only slightly more likely to value privacy and safety. However, given the small sample size of sub-groups, we would not place much weight on these differences.

## 4 Discussion

In this study, we carried out qualitative research into what people most valued about sanitation. We used the findings to develop a definition and conceptual model of sanitation-related quality of life in a low-income urban setting. Using attributes of “a good life” as an entry point for discussion, we identified five capabilities as core attributes of sanitation-related QoL: health, avoiding disgust, privacy, safety and avoiding shame. Our conceptual model outlines how improvements in sanitation commodities might improve capabilities via changes in these five attributes, and how capabilities are moderated by personal and environmental conversion factors.

Three aspects distinguish our study from previous work in this area, particularly studies focused on sanitation-related stress and insecurity (Caruso et al., 2017b; Kwiringira et al., 2014; Sahoo et al., 2015; Shiras et al., 2018). First, we conceptualise sanitation-related QoL as an outcome like health-related QoL, by contrast to insecurity and stress which are usually conceptualised as risk factors affecting mental health outcomes (Caruso et al., 2018). As a result, health is an attribute *within* the concept of sanitation-related QoL (Fig. 2). Second, we conceptualise sanitation-related QoL as applicable to the general population, while the literature on insecurity and stress has usually focused exclusively on women. Third, our approach to qualitative research was rooted in economics, in particular value and the capability approach.

We used pile-sorting and triads as alternatives to simple ranking in exploring relative value. The broad concurrence of the top-ranked attributes triangulated across methods gives us confidence in these findings. While concepts on pile-sorting cards were imposed on participants based on the literature, the corresponding advantage was that the participant's choice was direct, rather than the indirect interpretation of transcripts by the researcher.

Applying capability theory about conversion factors helps emphasise how two people achieving an identical improvement in objective toilet quality may experience a dissimilar QoL effect. Two people may experience the same toilet very differently. For example, because of social norms around gender, a middle-aged man might have a higher conversion factor than an adolescent girl, if she has different expectations of privacy and safety to achieve a given level of capability. His conversion factor might also be higher than an older man with restricted mobility who worries more about falling and finds it harder to avoid touching disgusting surfaces. This is important because many sanitation interventions and evaluations focus on access to a given technology or level of service, implicitly assuming it delivers similar benefits to all users.

Environmental conversion factors are also important. The social and environmental context in which a toilet commodity is used affects the capabilities an individual can derive from it. In several previous studies, convenience was identified as a valuable attribute of household toilets, as compared to open defecation or public toilets (Novotný et al., 2018). Our participants did not talk about convenience, since all used on-plot toilets and took this for granted. Similarly, it was unsurprising that water supply was rarely mentioned as an important influence on sanitation capabilities, contrary to some other settings (Sahoo et al., 2015), since all participants had a fairly reliable piped water supply within the compound.

Despite the differences in framing and methods, the five identified attributes are broadly consistent with studies of insecurity, stress and motives related to sanitation in both rural and urban areas, which is supportive of theoretical generalisability (Novotný et al., 2018; Sclar et al., 2018). One area of contrast relates to the ranking of attributes rather than their identification. “Fewer diseases”, as the card was framed, was consistently identified as the most valuable in pile-sorting and triads. It was also the most-frequently mentioned concept in interviews. In previous studies, disease prevention (or less often “health”, possibly in its broader sense) was typically ranked second or third, behind other attributes (Gross and Günther, 2014; Hulland et al., 2015; Jenkins, 1999; Jenkins and Curtis, 2005; Jenkins and Scott, 2007; Kiyu and Hardin, 1993; Lagerkvist et al., 2014; Mukherjee, 2001). The difference between first place and second or third place is not that great, but debate in the literature on the relative importance of disease prevention (Jenkins and Curtis, 2005) suggests that this merits discussion. We propose three possible explanations for disease prevention being the most valuable attribute of sanitation in this setting. First, all but one of the previous studies was in a predominantly rural setting. Our setting was urban, where populations generally have higher levels of education (Zhang, 2006) and greater exposure to media, which can influence health-related knowledge (Agüero and Bharadwaj, 2014; Yaya et al., 2018). Second, a participant identifying something as valuable or important is different to them identifying what motivates a specific behaviour (Aunger and Curtis, 2013). Therefore, given the majority of these previous studies were motives-oriented, they were measuring something slightly different to value. Third, half our interview sample comprised people who had received an NGO sanitation intervention a year or two previously, which included direct and indirect health messaging. This may have contributed to a real change in the relative value of attributes, or to social desirability bias if interviewers were perceived as linked to the intervention, despite being told this was not the case.

### 4.1 Limitations

Our findings reflect the setting in which the data were generated, namely a low-income area of urban Maputo where shared sanitation was common. This limits the transferability of our findings to other settings to some extent. However, since the attributes identified broadly align with studies in diverse countries under different disciplinary perspectives, a level of theoretical generalisability may be claimed. As with any interpretivist qualitative research based on conversational interaction, our findings describe only one version of reality. The majority of analysis was undertaken by a non-Mozambican researcher IR who may have misinterpreted some interaction in transcripts, though important or unclear passages were always discussed with ZA and the field team. The relatively small sample size precluded comparison of findings by sub-groups of gender or age. Likewise, only tentative conclusions can be drawn from the pile-sorting findings, particularly in relation to the differences in relative value attributed by women and men. Reasons for caution in interpreting the frequency data are not only the sample size, but also the fact that topic guides were flexible, with sanitation discussions guided by the QoL attributes respondents had mentioned in part 1. The fact that some participants had received an intervention, and others had not done so but were likely aware of the intervention in their area, may have biased their responses in unpredictable ways. For the 16% of interviews that involved some Changana, some meaning was possibly lost in translation to Portuguese, despite interviewers being fluent in both languages.

## 5 Conclusion

Our findings illustrate that people in low-income areas of Maputo, Mozambique, valued many different aspects of sanitation. Our interpretation of their accounts was captured in five core attributes of sanitation-related QoL: health, disgust, privacy, safety and shame. Our intention is to use these findings to inform the development of a quantitative measure in this setting, alongside quantitative methods of attribute valuation. We hope that others might explore sanitation-related QoL in other settings and populations to validate or develop the conceptual model. Sanitation interventions might improve different attributes of sanitation-related QoL to different degrees. The sixth sustainable development goal emphasises that sanitation for all should be adequate and equitable. Since two people might experience the same level of sanitation service very differently, thresholds of adequacy may differ across individuals and QoL effects of intervention may not be equitable. Future evaluations of sanitation interventions should consider how changes in quality of life might be captured, as well as changes in level of service and health outcomes.

## Funding

This work was supported by the 10.13039/501100000269Economic and Social Research Council through a PhD studentship. The fieldwork was funded by the 10.13039/100000865Bill & Melinda Gates Foundation. The funders had no role in the identification, design, conduct, or reporting of the analysis.

## Author contributions

Ian Ross: Conceptualisation, Methodology, Investigation, Formal analysis, Writing – original draft. Oliver Cumming: Supervision, Writing – review & editing, Funding acquisition. Robert Dreibelbis: Methodology, Writing – review & editing. Zaida Adriano: Methodology, Data curation, Investigation, Writing – review & editing. Rassul Nala: Writing – review & editing, Funding acquisition. Giulia Greco: Supervision, Methodology, Writing – review & editing.

## Declaration of competing interest

None.
