# Supplementary material for: How does sanitation influence people's quality of life? Qualitative research in low-income areas of Maputo, Mozambique
Source: Soc Sci Med. 2021 Mar;272:113709. doi: 10.1016/j.socscimed.2021.113709 (PMC7938219; doi:10.1016/j.socscimed.2021.113709)
Supplement: Multimedia component 1 [file mmc1.docx]

# Online supplementary materials

A. Reporting against COREQ checklist

B. Further information about the intervention

C. IDI topic guide

D. Additional findings

E. Original Portuguese of quotations

## A. Reporting against COREQ checklist

| **No.** | **Item** | **Guide questions/description** | **Page no.** | **Notes** |
| --- | --- | --- | --- | --- |
| **Domain 1: Research team and reflexivity** | | | | |
| **Personal Characteristics** | | | | |
| 1. | Interviewer / facilitator | Which author/s conducted the interview or focus group? | 6 | n/a – we worked with contracted interviewers with no interest in academic publication, due to Changana language requirements. |
| 2. | Credentials | What were the researcher's credentials? *E.g. PhD, MD* | n/a | Two interviewers had an undergraduate degree, and the other two a secondary education. |
| 3. | Occupation | What was their occupation at the time of the study? | n/a | Interviewers worked part-time alongside other various employment |
| 4. | Gender | Was the researcher male or female? | 6 | Two male, two female |
| 5. | Experience and training | What experience or training did the researcher have? | n/a | All had previous experience in social research, and two had experience facilitating focus groups. The week's training is described in the main body. |
| **Relationship with participants** | | | | |
| 6. | Relationship established | Was a relationship established prior to study commencement? | n/a | Individual interviewers had no established relationship with participants before the study and were from various parts of Maputo. [initials]’s firm have been undertaking MapSan data collection activities in these bairros since 2015. |
| 7. | Participant knowledge of the interviewer | What did the participants know about the researcher? e*.g. personal goals, reasons for doing the research* | n/a | We see no reason why participants would expect interviewers to have any personal goals related to the research. Reasons for the research were in the participant information sheet explained at the beginning of each data collection event. |
| 8. | Interviewer characteristics | What characteristics were reported about the interviewer/facilitator? e.g. *Bias, assumptions, reasons and interests in the research topic* | n/a | None. They introduced themselves as employees of [company]. Their clothes or manner of speaking may have led participants to judge their social status. |
| **Domain 2: study design** | | | | |
| **Theoretical framework** | | | | |
| 9. | Methodological orientation and Theory | What methodological orientation was stated to underpin the study? *e.g. grounded theory, discourse analysis, ethnography, phenomenology, content analysis* | 8 | Interpretivist position applying framework analysis with inductive open coding |
| **Participant selection** | | | | |
| 10. | Sampling | How were participants selected? *e.g. purposive, convenience, consecutive, snowball* | 6 | full detail at referenced location |
| 11. | Method of approach | How were participants approached? e*.g. face-to-face, telephone, mail, email* | 6 | full detail at referenced location |
| 12. | Sample size | How many participants were in the study? | 6 | See referenced location. nb. only one participant was sampled per compound. |
| 13. | Non-participation | How many people refused to participate or dropped out? Reasons? | n/a | Refusal to participate was 5% for interviews, which were arranged to suit the respondent, and 15% for focus groups, which took place on Saturday mornings. |
| **Setting** | | | | |
| 14. | Setting of data collection | Where was the data collected? e*.g. home, clinic, workplace* | n/a | Interviews took place in participants' home compounds during the week. Focus groups took place on Saturdays in community buildings, such as nurseries and offices of community organisations. |
| 15. | Presence of non-participants | Was anyone else present besides the participants and researchers? | n/a | For interviews, non-participants (particularly children) were occasionally within earshot. Focus groups took place without any non-participants present |
| 16. | Description of sample | What are the important characteristics of the sample? *e.g. demographic data, date* | Tab. 1 | full detail at referenced location |
| **Data collection** | | | | |
| 17. | Interview guide | Were questions, prompts, guides provided by the authors? Was it pilot tested? | 7 | full detail at referenced location and guide in supplementary material C |
| 18. | Repeat interviews | Were repeat interviews carried out? If yes, how many? | n/a | no, but the last 2 focus groups reconvened previous participants |
| 19. | Audio/visual recording | Did the research use audio or visual recording to collect the data? | 7 | Audio-recording. Any names or other identifiers included in audio recordings were redacted during transcription. All audio recordings were permanently deleted |
| 20. | Field notes | Were field notes made during and/or after the interview or focus group? | n/a | Interviewers took limited notes in interviews, in order to focus on the flow of the discussion. |
| 21. | Duration | What was the duration of the interviews or focus group? | 7-8 | full detail at referenced location |
| 22. | Data saturation | Was data saturation discussed? | 8 | full detail at referenced location |
| 23. | Transcripts returned | Were transcripts returned to participants for comment and/or correction? | n/a | no |
| **Domain 3: analysis and findings** | | | | |
| **Data analysis** | | | | |
| 24. | Number of data coders | How many data coders coded the data? | 8 | One coder. In addition, [initials] speaks Portuguese to a level sufficient for understanding original transcripts as well as translations. [initials] and [initials] discussed, with interviewers, the meanings of terms used by participants, to establish whether the way they were interpreted in English was the same as in Portuguese. |
| 25. | Description of the coding tree | Did authors provide a description of the coding tree? | Fig. 2 | The conceptual model comprises the final core attributes and underlying concepts |
| 26. | Derivation of themes | Were themes identified in advance or derived from the data? | 8 | derived from the data |
| 27. | Software | What software, if applicable, was used to manage the data? | 8 | nVivo 12 |
| 28. | Participant checking | Did participants provide feedback on the findings? | n/a | During the participant checking process in the last two focus groups, no substantial concerns or proposals were raised. |
| **Reporting** | | | | |
| 29. | Quotations presented | Were participant quotations presented to illustrate the themes / findings? Was each quotation identified? e*.g. participant number* | 10ff | full detail at referenced location |
| 30. | Data and findings consistent | Was there consistency between the data presented and the findings? | 19ff | full detail at referenced location |
| 31. | Clarity of major themes | Were major themes clearly presented in the findings? | Fig. 2 | full detail at referenced location |
| 32. | Clarity of minor themes | Is there a description of diverse cases or discussion of minor themes? | Fig. 2 | full detail at referenced location |

## B. Further information about the intervention

#### Types of toilets

The two types of toilets delivered as the MapSan trial intervention were, firstly, a single ‘shared toilet’ to be used by a minimum of 15 people, at 85% subsidy. The second is a community sanitation block to be used by a minimum of 21 people, at 90% subsidy. All households had on-plot piped water supply at the time of intervention, and CSBs have their own water tank on the roof. The NGO has been implementing the same intervention subsequent to MapSan in the same neighbourhoods. They have been iteratively improving variants of this intervention in low-income areas of Maputo since 2009. Photographs with typical examples of each of the three toilet types are below.

#### Pit latrines

| 1. Pit latrine with tyre and wood for squatting | 2. Pit latrine with concrete slab |
| --- | --- |
| 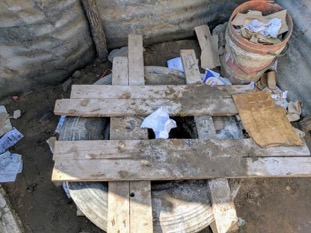 | 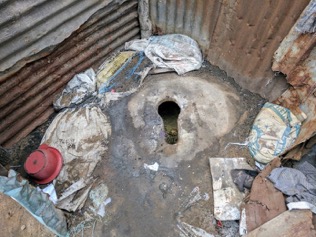 |
| 3. Fabric door providing limited privacy | 4. No door and adjacent greywater pit |
| 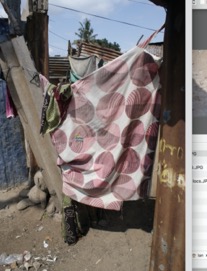 | 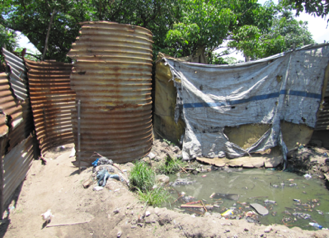 |

#### Shared toilets and community sanitation blocks

| **Exterior** | |
| --- | --- |
| 1. Shared toilet (ST) | 2. Community sanitation block (CSB) |
| 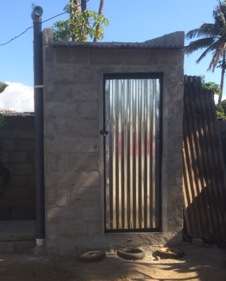 | 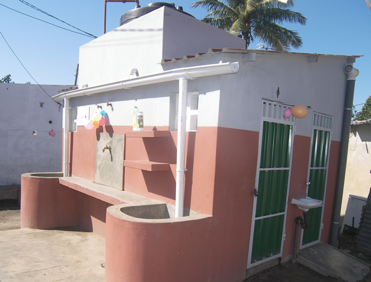 |
| **Interior (varied between CSB / ST depending on design)** | |
| 3. Squat pan | 4. Seat pan |
| 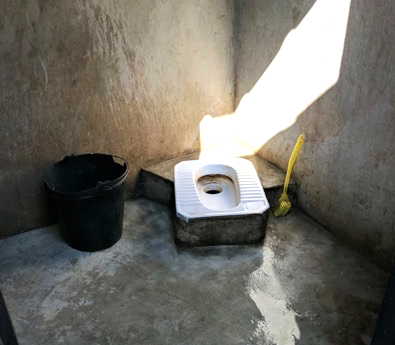 | 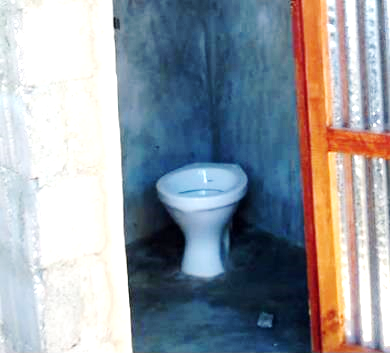 |

#### Further information about the intervention

We provide more information in Table A about the intervention, in the format of the TIDieR checklist (Hoffmann et al., 2014). More information about different aspect of the intervention is provided across various publications (Bick et al., 2020; Knee et al., 2020; Mattson, 2016).

Table A: Intervention description using TIDieR checklist

|  | **Item** | **Notes** |
| --- | --- | --- |
| **1** | Provide the name or a phrase that describes the intervention. | Subsidised pour-flush toilets shared by multiple households |
| **2** | Describe any rationale, theory, or goal of the elements essential to the intervention. | In this setting there is limited space and willingness or ability to pay for private toilets, and households already use low-quality shared pit latrines. |
| **3** | Materials: Describe any physical or informational materials used in the intervention, including those provided to participants or used in intervention delivery or in training of intervention providers. Provide information on where the materials can be accessed (e.g. online appendix, URL). | The intervention provided two types of toilet facility (photos above), alongside education on their use and maintenance. There were also two hygiene promotion visits after completion of construction, carried out by paid staff who received 2 days of training. These focused on contamination routes, good personal hygiene practice, and handwashing with soap. More information is provided elsewhere (Bick et al., 2020; Knee et al., 2020; Mattson, 2016). |
| **4** | Procedures: Describe each of the procedures, activities, and/or processes used in the intervention, including any enabling or support activities. | Key procedures included:   1. Community engagement and site identification – undertaken by eight contracted community-based organisations (CBOs), e.g. assessment of demand for better toilets and localised environmental issues affecting site selection (e.g. water table) 2. Site selection and preparation – site selection undertaken by WSUP in discussion with CBOs, and site preparation (e.g. emptying of old latrine pits) undertaken by contracted firms. 3. Toilet construction – undertaken by contracted construction firms 4. Education on use, maintenance and hygiene – undertaken by contracted ‘sanitation activists’ |
| **5** | For each category of intervention provider (e.g. psychologist, nursing assistant), describe their expertise, background and any specific training given. | Main stakeholders in delivery included:   1. **Water and Sanitation for the Urban Poor** (international NGO) – overall lead on intervention delivery. Team included engineers and community engagement specialists. 2. **Various community-based organisations** – sub-contractor facilitating community engagement. 48 people trained. Teams included facilitators from the local area of the intervention. 3. **Various construction firms** – Sub-contractors building the toilet infrastructure. They were predominantly small local firms. 4. **Sanitation activists** – Sub-contractors educating toilet users and promoting hygiene. 55 people trained. 5. **Municipality and World Bank** – oversight and approvals. Team included engineers. |
| **6** | Describe the modes of delivery (e.g. face-to-face or by some other mechanism, such as internet or telephone) of the intervention and whether it was provided individually or in a group. | All engagement was face-to-face. As this was shared sanitation, any site visits were made to compound members jointly, rather than individually. |
| **7** | Describe the type(s) of location(s) where the intervention occurred, including any necessary infrastructure or relevant features. | Setting described fully in manuscript main body. |
| **8** | Describe the number of times the intervention was delivered and over what period of time including the number of sessions, their schedule, and their duration, intensity or dose. | All aspects of the intervention delivered only once, except for two hygiene promotion visits. |
| **9** | If the intervention was planned to be personalised, titrated or adapted, then describe what, why, when, and how. | n/a |
| **10** | If the intervention was modified during the course of the study, describe the changes (what, why, when, and how). | n/a |
| **11** | Planned: If intervention adherence or fidelity was assessed, describe how and by whom, and if any strategies were used to maintain or improve fidelity, describe them. | n/a |
| **12** | Actual: If intervention adherence or fidelity was assessed, describe the extent to which the intervention was delivered as planned. | Fidelity was assessed by Bick et al. (2020) |

## C. Interview topic guide

#### Part 1. What is a good life? (15 mins)

We would like to talk about things that are important for a good life, such as:

- things you can buy and touch (for example, food)
- things you cannot buy (for example, family)
- things you can feel but cannot touch (for example, happiness)

What do you think is important for a good life?

Think about people who live in this neighbourhood who have a good life.

List ten things that make these people have a good life.

*[probe – remind about things you cannot buy or touch]*

Now think about people who live in this neighbourhood who have a bad life:

*[probe – don’t just think about the opposites of things for a good life]*

#### Part 2. Contribution of sanitation to a good life (20-40 mins)

Let's talk about toilets and sanitation.

What types of toilets do people use in this neighbourhood?

*[note – keep this short, just to establish that there are many types]*

Earlier you made a list of things important for a good life.

Now let's talk about how good sanitation and good toilets contribute to each of these things. They can also be examples of bad toilets or sanitation.

Think about all things you use the toilet for.

Let’s talk about [*QoL card X*] that you mentioned.

*[QoL cards include: Enough food, Education for children, Security, Housing, Happy family and children, Partner, Friends / neighbours, Physical health, Mental health, Clean environment, Water and Sanitation, Good work/job, Enough money to live, Freedom / independence, Political voice, Happiness, Being respected]*

How do you think that using good or bad toilets affect [*QoL card X*]?

It can be positive, negative, or maybe the answer is "not at all".

Think about how the effect can be different for people of different ages and sexes.

Now let's consider [*QoL card Y*]

*[continue until all cards have been addressed, or until you run out of time]*

*[If the conversation is not flowing, pick a random QoL card which they didn’t mention, emphasising that these are what others said was important. Alternatively, ask questions such as “are you satisfied with the type of bathroom you are using now?” or “If you improved your toilet, how would your quality of life change?”]*

Can you think of other ways that good toilets contribute to a good life, that are not on a card?

#### Part 3. Pile-sorting component (5-10 mins)

These are things that other people said were important about sanitation.

You have already mentioned some of them.

Let’s classify them according to their importance to live a good life.

First, select the five things that are the most important for a good life.

*[probe - why did you make that choice? What makes these things more important?]*

Now, select the five things which are next most important.

*[probe - why did you make that choice? What makes these things more important?]*

Close

An example of a pile-sorting card is provided below – the label reads “Not smelling faeces”. The 15 cards comprise the bar labels in Figure 4.

| **Não cheirando fezes**  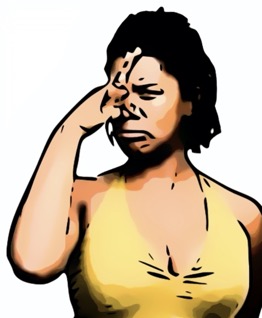 |
| --- |

## D. Additional findings

#### Matching concepts from the literature onto attributes

The identified attributes align to a great extent with the 15 concepts used for the pile-sorting cards identified from the literature (Table A). As noted in relation to Figure 4, concepts which do not fit under any single attribute scored lower in pile-sorting than the highest-ranked concepts of our five core attributes, and were in the bottom 40% of cards overall. Notes on these pile-sorting concepts are provided in Table B.

Table B: How pile-sorting concepts from the literature match onto identified attributes

| **Identified attribute** | **Concept on pile-sorting card from literature** |
| --- | --- |
| Health | Fewer diseases |
| Disgust | Seeing faeces less |
|  | Better personal cleanliness |
|  | Fewer flies |
|  | Smelling faeces less |
| Shame | More dignity |
|  | More pride |
|  | Less shame when visitors come |
| Safety | More safety for women and girls |
| Privacy | More privacy |

Table C: Notes on pile-sorting concepts which do not map onto a specific attribute

| **Concept on pile-sorting card from literature** | **Notes on why it does not map under any single attribute** |
| --- | --- |
| More comfort | Depending on the way the concept is used, the associated feeling or experience could conceivably be included under any of the five attributes. For example:   - disgust, e.g. feeling more comfortable or at ease when not seeing, smelling or touching disgusting things - safety, e.g. feeling more comfortable or at ease when not worried about having an accident or being assaulted |
| Cleaner environment | As distinct from disgust related to specifically using the toilet, or its direct local consequences (e.g. overflowing latrines), the concept of a clean environment is much broader. When mentioned by participants, it was sometimes framed in terms of solid waste. In terms of sanitation, it relates more to environmental conditions arising from excreta in floodwater or illegal dumping of faecal sludge, rather than sanitation behaviours within the household. Therefore, the concept of a clean environment would either be included under disgust or not be a part of SanQoL as defined in this paper. |
| More convenience | See discussion section of manuscript |
| Easier for people with restricted mobility | In capability terms, restricted mobility is a conversion factor. Therefore, depending on the way the concept is used, the associated feeling or experience could conceivably be included under any of the five attributes. For example:   - disgust, e.g. being less able to avoid seeing, smelling or touching disgusting things due to restricted mobility - shame, e.g. being ashamed or feeling less dignified as a result of being less able to carry out sanitation practices than one would like |
| Reduced conflict with neighbours | Depending on the way the concept is used, the associated feeling or experience could be included under:   - shame, e.g. if conflict about sanitation arises from a behaviour or its consequence considered shaming - disgust, e.g. if conflict arises because the toilet is disgusting neighbours within or outside the compound |

#### Triads methods and results

**Triads methods**

Participants were shown a set of nine A4 cards, each with an attribute of sanitation-related QoL identified as most important in emerging analysis of transcripts. A sub-set of three cards was then read out by an interviewer and placed on the floor for all to see. Participants were asked to choose the attribute they considered the most important of the three, and to tick the relevant box using a sheet and pen provided. The vast majority of participants were literate, but interviewers (two per group) supported the few who were not. This was repeated 15 times with different combinations of attributes. For the analysis, concepts were allocated to three groups based on the frequency with which they were preferred. The same scoring as IDIs was then applied (first group: 3 points, second: 1 point, third: 0 points).

The attributes on the triad cards were slightly different to the pile sorting attributes, for two reasons. First, it would have risked participant fatigue to include all 15 pile-sorting attributes in triadic comparison with one another in multiple possible combinations. We settled on nine triad attributes as providing the optimal balance. Second, the pile sorting attributes were based on the literature, and we thought it better to update them based on emerging findings from interviews and focus groups.

**Triads results**

The triad exercise in the final two FGDs generated 195 observations. Results were broadly similar to the pile-sorting, except concepts linked to shame scored higher. "Managing children's faeces easily" was included as a sense-check as a concept that was being mentioned in emerging analysis. "Harmony on sharing cleaning responsibilities" was more important in transcripts and scored especially high amongst women (who were more likely to carry out cleaning).

Figure A: Triadic comparison of attributes during 2 x FGDs (n=13 respondents)

|  |
| --- |
| *nb. labels (without bracketed part) were written on A4 cartoon cards in Portuguese and read out by interviewers. n/a = not applicable.* |

## E. Original Portuguese of quotations

Original Portuguese of quotations used in text.

| English | Portuguese |
| --- | --- |
| **Health** | **Saúde** |
| “You feel under pressure when the bathroom is dirty, and you don’t feel at ease.” Female FGD, 18-24 (FGF01) | "Sentes um aperto quando a casa de banho fica suja e não se sentes a vontade" |
| “It is difficult for us to control what children do. An adult knows they shouldn’t touch something, or they'll catch germs, but a child doesn't know.” Male IDI, 36 (EAGJ04) | "é difícil nós controlarmos as crianças, o sitio onde um adulto sabe que não posso pegar aqui senão vou ter micróbios a criança não sabe" |
| “When it rains the faeces in the pit rise up, then we get diseases because cholera comes from there” Female IDI, 71 (EANC04) | "Quando chove as fezes sobem e de repente te deparas com aquelas larvas nesse momento traz doenças dentro de casa como cólera" |
| “Having a good toilet contributes positively to all these aspects, mental health, wellbeing for the soul, and general health as a whole.” Male IDI, 64 (EAGJ03) | “ter uma boa casa de banho contribui positivamente para todos esses aspetos que me referia, saúde mental, contribui para o bem estar para a alma, contribui para a saúde em geral no seu todo” |
| “Your neighbours will know the origin of the smell and will start to talk about it, and you can’t feel relaxed.” Female IDI, 76 (EAET04) | “vão saber a origem do cheiro e os vizinhos irão começar a falar e isso não te pode deixar tranquila” |
| **Disgust** | **Nojo** |
| "It is something so horrible to see other people's faeces." Male IDI, 19 (EAGJ02) | "é algo tão horrivel estar a ver fezes de outras pessoas" |
| “You cannot eat because you lose your appetite … due to the smell. You don’t even feel free to come out of your house because it smells bad out there.” Female FGD, 25-59 (FGF02) | “Mesmo comer você não pode, porque perde apetite … devido ao cheiro. Nem tem vontade de sair de dentro de casa porque fora cheira mal.” |
| “[In the toilet] I would feel like I am in the kitchen. With no bad smell, it seems like you could even drink tea in there, without realising you are in a toilet.” Female IDI, 71 (EANC04) | "Parece que estou a entrar na cozinha, não cheira nem nada até parece que vais entrar na casa de banho para tomar chá sem se aperceber que ali é uma casa de banho" |
| When the house is clean but the toilet is not, this is undignified.” Female FGD, 60+ (FGF03) | “quando a casa fica limpa e a casa de banho não, não traz dignidade.” |
| **Shame** | **Vergonha** |
| “A person's toilet becomes the mirror of that person.” Male IDI, 64 (EAGJ03) | "A casa de banho da pessoa torna-se o espelho da pessoa" |
| “Everyone will refer to you according to the state of your toilet, saying ‘it's there at her house that the toilet smells’ … nobody respects you.” Female IDI, 27 (EAET05) | "Toda gente irá referenciar te em função do estado da sua casa de banho, dizendo é ali em casa da dona *[name redacted]* que cheira casa de banho … ninguém te respeita." |
| “People who go down my road smell the stench from my toilet. Then when they later pass me on the street they will look at me in a different way.” Male IDI, 19 (EAGJ02) | "Quem passa da minha rua e ao lado da minha casa sentisse o meu mau cheiro que parte da casa de banho da minha casa, essa pessoa, no dia que for a cruzar-se comigo na rua epah, me repararia de uma outra forma" |
| “When I get visitors, I can let the person use the toilet without fear. I think this makes people look at me differently, with respect.” Male IDI, 28 (EAJP05) | "Recebo visitas, posso muito bem deixar que a pessoa use a casa de banho sem receio, então acho que isso acaba fazendo com que as pessoas olhem-me de outra forma, com respeito" |
| “If a visitor asks to go to the toilet and sees it in good condition, they’ll say ‘wow, that lady’s house is hygienic’” Female FGD, 60+ (FGF03) | “Chegar alguém e pedir para ir a casa de banho, e quando chega lá, percebe que está em condições. Na saída dele vai dizer ‘hiii em casa de fulano há higiene’” |
| Safety | **Segurança** |
| “There are people who are raped while they use these toilets. … there are times we even have to defecate in a bucket because we fear bandits.” Female IDI, 27 (EAET05) | "Existem pessoas que são violadas por causa de usarem estas casas de banho, é por isso que a noite usamos baldinhos para o efeito, temendo violência" |
| “that toilet built from car tyres is a hazard – when it rains it could come crashing down at any moment.” Male FGD, 25-59 (FGM02) | "Aquele pneu é um risco a qual, quando chove a qualquer momento aquilo pode desabar." |
| “I’m afraid to use it at night because I wouldn’t know which way to enter, where to tread inside, and I would be afraid of falling into the hole.” Female IDI, 71 (EANC04) | "Não posso ter coragem porque ao sair de noite não saberia como entrar, onde pisar dentro da casa de banho e tenho medo de entrar ali no buraco" |
| Privacy | **Privacidade** |
| “While you walk to work, … you might see a woman with just a bit of capulana [fabric], when she is naked taking a bath.” Male IDI, 28 (EAJP05) | "A gente quando vai ao trabalho, ... dá para perceber uma mulher têm um bocado de capulana, quando é que está despida no banho" |
| “When a bathroom is not secure you do not feel free to use it, because at any moment an individual can enter.” Female IDI, 76, (EAET04) | "Quando uma casa de banho não oferece segurança voce não esta livre de utiliza-la porque a qualquer momento poderá entrar um individuo dentro" |
| “You cannot imagine the gymnastics I do when I have my period. I do not feel relaxed because I do not know if I'm being watched.” Female IDI, 27 (EAET05) | "Não imaginas a ginástica que eu faço quando estou nos dias da minha menstruação, não me sinto a vontade porque não sei se estou sendo observada" |

## References in online supplementary materials

Bick, S., Buxton, H., Chase, R., Ross, I., Adriano, Z., Capone, D., … Dreibelbis, R. (2020). Using path analysis to test Theory of Change: a quantitative process evaluation of the MapSan trial. *Under Review*.

Hoffmann, T. C., Glasziou, P. P., Boutron, I., Milne, R., Perera, R., Moher, D., … Michie, S. (2014). Better reporting of interventions: template for intervention description and replication (TIDieR) checklist and guide. *BMJ : British Medical Journal*, *348*, g1687. https://doi.org/10.1136/bmj.g1687

Knee, J., Sumner, T., Adriano, Z., Anderson, C., Bush, F., Capone, D., … Brown, J. (2020). Effects of an urban sanitation intervention on childhood enteric infection and diarrhoea in Mozambique. *MedRxiv Preprint*. https://doi.org/https://doi.org/10.1101/2020.08.20.20178608

Mattson, K. (2016). Final Evaluation of the Water & Sanitation for the Urban Poor JSDF Funded Maputo Peri-Urban Sanitation Project.
